# Study of the Effect of Doping ZrO_2_ Ceramics with MgO to Increase the Resistance to Polymorphic Transformations under the Action of Irradiation

**DOI:** 10.3390/nano11123172

**Published:** 2021-11-23

**Authors:** Alisher E. Kurakhmedov, Mahambet Alin, Adilet M. Temir, Igor A. Ivanov, Yeugeniy V. Bikhert, Yerulan O. Ungarbayev, Maxim V. Zdorovets, Artem L. Kozlovskiy

**Affiliations:** 1 Laboratory of Solid State Physics, The Institute of Nuclear Physics, Almaty 050032, Kazakhstan; a.kurahmedov@inp.kz (A.E.K.); a.temir@inp.kz (A.M.T.); igor.ivanov.inp@gmail.com (I.A.I.); e.bikhert@inp.kz (Y.V.B.); e.ungarbayev@inp.kz (Y.O.U.); mzdorovets@gmail.com (M.V.Z.); 2 Engineering Profile Laboratory, L.N. Gumilyov Eurasian National University, Nur-Sultan 010008, Kazakhstan; kazpost93@gmail.com; 3 Department of Intelligent Information Technologies, Ural Federal University, 620075 Yekaterinburg, Russia; 4 Institute of Geology and Oil and Gas Business, Satbayev University, Almaty 050032, Kazakhstan

**Keywords:** ZrO_2_ ceramic, swift heavy ion, polymorphic transformations, radiation defects, radiation resistance

## Abstract

The purpose of this study is to assess the effect of doping ZrO_2_ ceramics with MgO on radiation swelling and polymorphic transformations, as a result of irradiation with heavy ions. Interest in these types of materials is due to the great prospects for their use as structural materials for new-generation reactors. The study established the dependences of the phase composition formation and changes in the structural parameters following a change in the concentration of MgO. It has been established that the main mechanism for changing the structural properties of ceramics is the displacement of the cubic c-ZrO_2_ phase by the Zr_0.9_Mg_0.1_O_2_ substitution phase, which leads to an increase in the stability of ceramic properties to irradiation. It has been determined that an increase in MgO concentration leads to the formation of an impurity phase Zr_0.9_Mg_0.1_O_2_ due to the type of substitution, resulting in changes to the structural parameters of ceramics. During studies of changes in the strength properties of irradiated ceramics, it was found that the formation of a phase in the Zr_0.9_Mg_0.1_O_2_ structure leads to an increase in the resistance to cracking and embrittlement of the surface layers of ceramics.

## 1. Introduction

In light of the latest world trends in energy development, increasing attention is paid to nuclear and atomic energy in view of their potential use not only as a basis for energy production but also—in the case of nuclear reactors—hydrogen production [1,2,3]. In this regard, research into new types of structural materials for the high-temperature nuclear reactors of a new generation deserve special attention. The most important requirements for new types of structural materials for Gen IV reactors include high melting points, thermal conductivity, resistance to radiation damage, etc. [4,5,6]. According to the shortlist for the EUROfusion project, alternatives to traditional steels and alloys that meet all of the above requirements are ceramic materials based on oxides (ZrO_2_, BeO, MgO) [7,8,9,10], nitrides (AlN, Si_3_N_4_) [11,12,13], and carbides (SiC, TaC) [14,15]. Among the various types of ceramics, oxide ceramics ZrO_2_, BeO have recently been recognized. With a small value of the thermal neutron capture cross section, good corrosion and degradation resistance, as well as excellent strength and hardness indicators, there are broad prospects for their use as the structural materials of a new generation [16,17,18,19,20].

Interest in ZrO_2_ ceramics is also due to the fact that, when used as materials for fuel assemblies (FAs), ceramics create an additional protective barrier for oxygen and hydrogen penetration from the coolant, thereby reducing the rate of FA degradation. However, a number of studies show that in ZrO_2_ ceramics, under the action of irradiation with heavy ions of Kr, Xe at high irradiation fluences, polymorphic transformations of the t-ZrO_2_ → c-ZrO_2_ type occur, caused by the processes of radiation damage [21,22,23]. Such transformations can have a negative impact on resistance to degradation, hydrogen absorption, and swelling of ceramics as a result of long-term operation. The mechanisms of polymorphic transformations are primarily associated with local thermal effects arising from the interaction of ions with the irradiated material, and the subsequent transformation of the ion’s kinetic energy into thermal energy. As a result of such interactions, nonequilibrium regions appear in the structure, leading to phase transformations. Moreover, as shown in [21,22], the degree of polymorphic transformation depends largely on the energy and types of ion with which the interaction occurs.

One way to increase resistance to the polymorphic transformations of oxide and nitride ceramics is to introduce various stabilizers into their structure, which, due to their properties, can interfere with these processes [24,25]. The most common stabilizing additives are Y_2_O_3_, MgO and Al_2_O_3_, which, when added in small amounts, lead to significant changes in the properties of ceramics [26,27,28,29,30].

Based on the above, the aim of this work is to study the effect of MgO addition at concentrations of 0.05, 0.10 and 0.15 mol.% during sintering of ZrO_2_ ceramics on the increase in resistance to polymorphic transformations t-ZrO_2_ → c-ZrO_2_ under the action of irradiation.

## 2. Materials and Methods

The samples for the study were ZrO_2_ ceramics doped with MgO. Preparation of the samples consisted of weighing the initial powders of ZrO_2_ and MgO in specified molar ratios and grinding them in a planetary mill for 1 h at a grinding speed of 400 rpm. After grinding, the samples were sintered at a temperature of 1100 °C for 5 h and then pressed into tablets with a diameter of 5 mm and a thickness of 200 μm.

The concentrations of the MgO dopant were 0.05, 0.10 and 0.15 mol.%; these values were chosen with a view to studying the effect of additives on changes in structural and strength properties. Micron-sized ZrO_2_ and MgO powders manufactured by Sigma Aldrich (Sigma Ltd., Saint Louis, MO, USA) were used as starting reagents; the chemical purity was 99.95%.

Assessment of the resistance of samples to radiation damage was carried out by irradiating them with heavy Xe^22+^ ions with an energy of 230 MeV and fluences of 10^12^–10^15^ ion/cm^2^. Irradiation was carried out at room temperature, with the ion flux at 10^9^ ion/cm^2^*s, and the beam current was 100 nA. The ion type and energy values were chosen with a view to simulating radiation damage, comparable to the damage arising from irradiation with uranium fission fragments in reactor tests. The values for irradiation fluences were chosen with a view to simulating defects, both in the case of single defects and in the overlap of defect regions formed by the passage of ions in the ceramic material. According to the calculated data, the energy losses of incident Xe^22+^ ions are dE/dx_electron_ = 24,700 keV/μm, dE/dx_nuclear_ = 86.5 keV/μm, and the ion path length is more than 14 μm.

The study of the kinetics of polymorphic transformations as a result of irradiation and subsequent changes in structural parameters was carried out using data obtained from X-ray diffraction. The data acquisition was carried out on a D8 Advance ECO X-ray diffractometer (Bruker, Mannheim, Germany). Diffraction patterns were recorded using Cu-kα X-rays with a wavelength of 1.54 Å, with the Bragg–Brentano geometry in the angular range of 2θ = 25–75°, and a step of 0.03°. The crystal lattice parameters were determined and refined using the DiffracEVA v. 4.2 program code (Bruker, Mannheim, Germany), which is based on refinement of the parameters of the full-profile analysis methods.

To determine the phase composition, the PDF-2(2016) database was used. The phase ratio was determined using the formula $V_{\mathrm{admixture}}=\frac{RI_{\mathrm{phase}}}{I_{\mathrm{admixture}}\;+\;RI_{phase}}$, where *I_phase_* and *I_admixture_* are the intensities of the reflections of the dominant and impurity phases, and *R* = 1.45 is the structural parameter [31].

Structural deformation was evaluated by comparing changes in crystal lattice parameters depending on the types of external effects.

The swelling of the crystal structure was established by determining the value of Δ*V*/*V*—the change in the volume of the crystal lattice depending on the types of external effects.

Determination of strength properties was carried out by evaluating changes in the hardness of the ceramics before and after irradiation, as well as by calculating the degree of softening and degradation of the near-surface layer, depending on the irradiation fluence. The hardness was determined by an indentation method using a Vickers diamond pyramid at an indenter load of 500 N. The destruction degree was determined by calculating the ratio of hardness readings in the initial and irradiated states.

## 3. Results and Discussion

**Figure 1:**
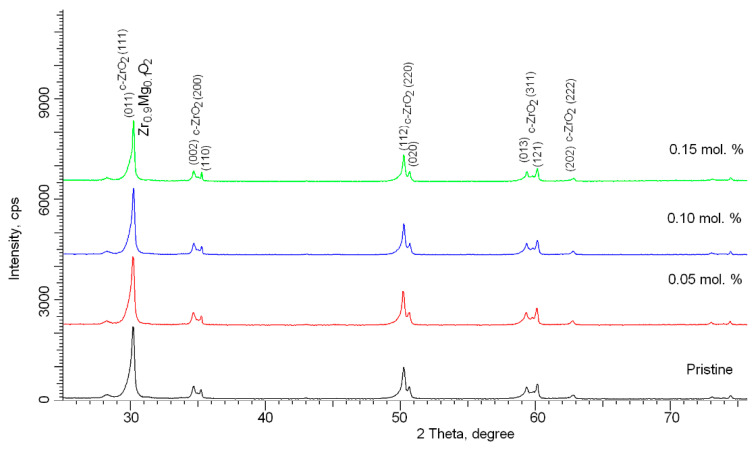
X-ray diffraction patterns of the studied samples of ceramics depending on the concentration of the MgO dopant.

Figure 1 shows the results of X-ray analysis of the samples under study, depending on the concentration of the dopant after thermal sintering. An initial sample without the addition of the dopant MgO, milled under the same conditions and subjected to thermal annealing, was chosen as a reference sample. According to the full-profile analysis data, in the initial state, the samples are a mixture of two phases, tetragonal (t-ZrO_2_) and cubic (c-ZrO_2_), the content of which is t-ZrO_2_/c-ZrO_2_~85/15. This phase composition is due to the conditions for obtaining ceramics and is a result of mechanochemical treatment and subsequent thermal sintering. The addition of the MgO dopant at a concentration of 0.05 mol.% does not lead to the formation of new reflections, which indicates the absence of phase transformation processes and the formation of substitution or interstitial phases. However, in the case of an increase in concentration to 0.10 mol.% and higher, the general broadening of the main reflections can be described by three sets of functions, with maxima related to the t-ZrO_2_ and c-ZrO_2_ phases, and maxima characteristic of the cubic substitutional phase of Zr_0.9_Mg_0.1_O_2_. Evaluation of the contributions of these phases to the composition of ceramics showed that the content of this phase is 3–5%, while partially replacing the c-ZrO_2_ phase. The formation of the Zr_0.9_Mg_0.1_O_2_ phase with an increase in dopant concentration is due to the substitution of magnesium atoms with zirconium atoms, followed by the formation of a cubic lattice. Thus, from the obtained X-ray phase analysis data, it can be concluded that when the dopant MgO is added with a concentration of more than 0.10 mol.%, under the selected synthesis conditions, a stable substitution phase is formed with a subsequent increase in its content. However, when analyzing the diffraction pattern of a sample with an MgO dopant content of 0.15 mol.%, it was found that an increase in the content of the Zr_0.9_Mg_0.1_O_2_ phase leads to a strong deformation of the peaks, which is characteristic of distortions of interplanar spacings and distortion of the crystal lattice.

**Figure 2:**
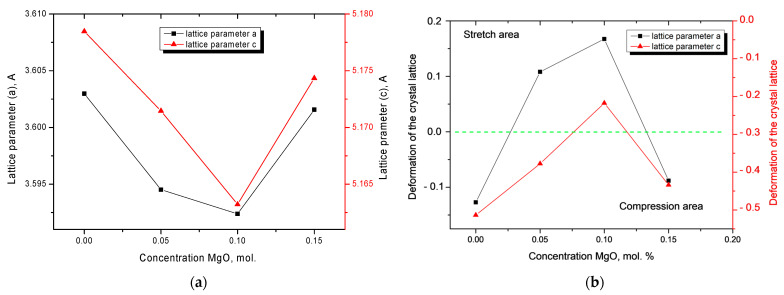
(**a**) Changes in the crystal lattice parameters depending on the MgO concentration; (**b**) deformation of the crystal lattice depending on the MgO concentration.

Figure 2 shows the results of changes in the crystal lattice parameters depending on the concentration of the MgO dopant. According to the data obtained, an increase in dopant concentration from 0.05 to 0.10 mol.% leads to a decrease in crystal lattice parameters. This behavior of changes in parameters can be caused by partial substitution of a Zr atom with Mg atoms, which leads to a decrease in the cell size, as well as the formation of an impurity substitution phase. At the same time, the change in the lattice parameter *c* is more pronounced than the lattice parameter *a*. However, an increase in the MgO concentration to 0.15 mol.% leads to an increase in the crystal lattice parameters; this may be associated with an increase in the contribution of the Zr_0.9_Mg_0.1_O_2_ phase, which leads to deformation of the structure.

Analysis of the dependences Δ*a*/*a* and Δ*c*/*c* presented in Figure 2b reflect the deformation of compression or expansion of the lattice, depending on external factors. As seen in the data obtained, a change in the phase composition leads to an ordering of the crystal lattice and a decrease in tensile strain. However, an increase in the contribution of the Zr_0.9_Mg_0.1_O_2_ phase at a dopant concentration of 0.15 mol.% leads to the appearance of additional distortions of the structure, which negatively affects the changes in the crystal lattice.

One of the features of ZrO_2_ ceramics in the tetragonal phase is its instability as a result of external influences associated with the occurrence of thermal heating or deformation of the structure. This instability is expressed in a polymorphic transformation of the t-ZrO_2_ → c-ZrO_2_ type, accompanied by changes in the strength and structural properties of ceramics. As shown in [21,22], one of the mechanisms of polymorphic transformations is the thermal effect, arising from the transition of incident ions’ kinetic energy into thermal energy, as a result of energy loss along their trajectory in the material. At the same time, there is an increase in the irradiation fluence, followed by an increase in the effect of overlapping defect regions, which arise as a result of the overlapping trajectories of ions in the material; this leads to an increase in the thermal effect, which causes the transformation of the t-ZrO_2_ → c-ZrO_2_ structure. The result of this enhancement was the complete transformation t-ZrO_2_ → c-ZrO_2,_ with a subsequent deterioration in the structural and strength properties of ceramics. Moreover, as shown in [21,22], there is a direct dependence of the degree of polymorphic transformations not only on the irradiation fluence but also on the types of ion and their energy.

One method of controlling this effect in oxide ceramics is the use of dopants, the main role of which is to stabilize the structure and inhibit the mechanisms of polymorphic transformations. The basis of the braking mechanisms is the assumption that the introduction of a dopant with the subsequent substitution of zirconium atoms in the lattice nodes, as well as the formation of substitution phases, can lead to the creation of additional barriers and drains of defects.

**Figure 3:**
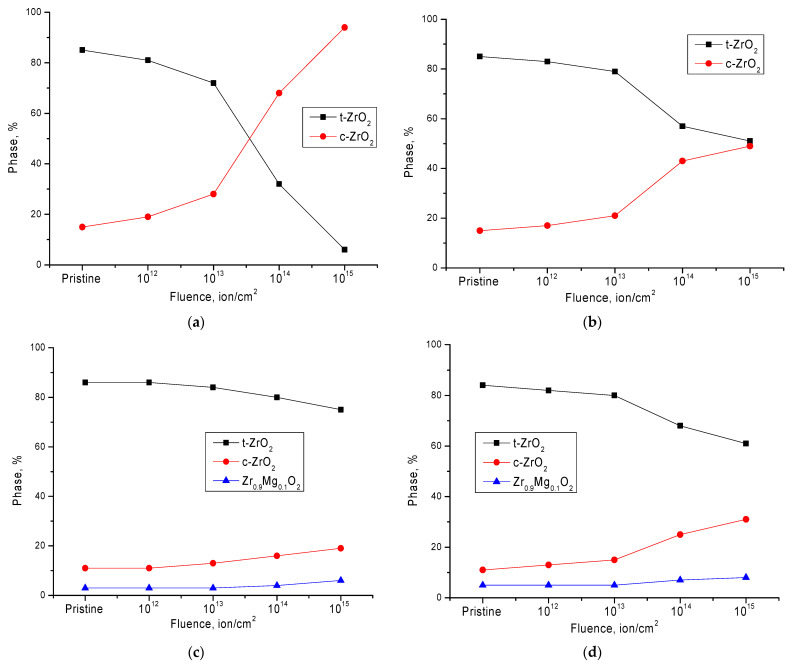
Diagrams of phase polymorphic transformations in ceramics as a result of irradiation with heavy ions: (**a**) initial sample; (**b**) doped with 0.05 mol.% MgO; (**c**) doped with 0.10 mol.% MgO; (**d**) doped with 0.15 mol.% MgO.

Figure 3 below shows the results of changes in the phase composition of the studied ceramics depending on the irradiation fluence, as well as the concentration of the dopant in the structure. In the case of initial, undoped ceramics, the dynamics of changes in the phase composition as a result of polymorphic transformations t-ZrO_2_ → c-ZrO_2_ are in good agreement with the results of studies performed on commercial ceramics [21,22]. In this case, the main process of polymorphic transformations, followed by the dominance of the c-ZrO_2_ phase, occurs at an irradiation fluence above 10^13^ ion/cm^2^; this is typical for the appearance of overlapping defect regions and, consequently, an increase in distorting stresses, as well as the thermal effect during irradiation.

In the case of doped ceramics, the effect of polymorphic transformation as a result of irradiation is less pronounced. Thus, at a dopant concentration of 0.05 mol.%, an increase in the irradiation fluence above 10^13^ ion/cm^2^ leads to an increase in the contribution of the c-ZrO_2_ phase, but the t-ZrO_2_ phase remains dominant. The formation of the Zr_0.9_Mg_0.1_O_2_ phase in the ceramic structure, as seen in Figure 3c, leads to a decrease in the mechanism of polymorphic transformations and an insignificant decrease in the t-ZrO_2_ phase. It should be noted that at an irradiation fluence of 10^14^–10^15^ ion/cm^2^, there is an insignificant increase in the contribution of the Zr_0.9_Mg_0.1_O_2_ phase, which inhibits polymorphic transformation of the t-ZrO_2_ → c-ZrO_2_ type.

At the same time, the presence of the Zr_0.9_Mg_0.1_O_2_ phase in the initial composition with a content of more than 5% leads to the destabilization of the ceramic structure and the acceleration of the process of polymorphic transformations at high irradiation fluences.

**Figure 4:**
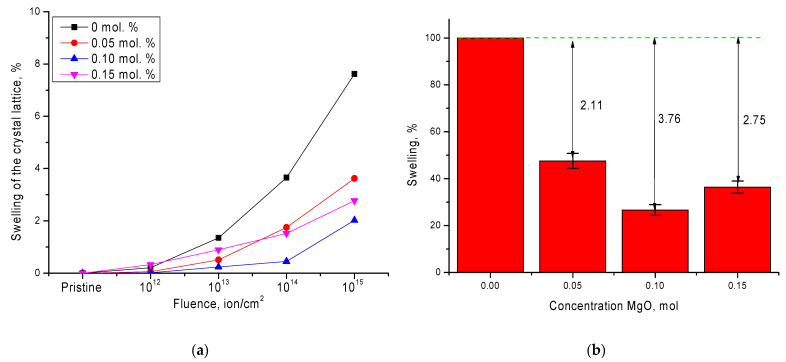
(**a**) Swelling of the crystal lattice of ceramics depending on the irradiation fluence; (**b**) comparative diagram of the swelling resistance of doped ceramics at maximum irradiation fluence (10^15^ ion/cm^2^).

The change in structural parameters as a result of phase polymorphic transformations is primarily associated with the deformational nature of the distortions, as well as swelling of the crystal structure. The results of assessing the degree of swelling, as well as the effect of doping on swelling resistance, are shown in Figure 4. As seen in the data presented, the greatest swelling of the crystal structure is observed in the case of undoped ceramics and at the maximum irradiation fluence is more than 7.5%. This behavior of the crystal lattice is primarily due to structural distortions resulting from polymorphic transformations, as well as the accumulation of radiation damage as a result of irradiation. Doping of ceramics leads to a more than twofold decrease in the degree of swelling at a dopant concentration of 0.05 mol.%, decreasing by a further 2.7–3.7% when the impurity phase Zr_0.9_Mg_0.1_O_2_ is formed in the structure, which reduces the degree of polymorphic transformations.

One of the important indicators of resistance to external influences—in particular, irradiation with heavy ions and subsequent accumulation of radiation damage—is the preservation of the strength properties of oxide ceramics. This is because the mechanical properties are very sensitive to radiation defects [32,33]. In this case, the presence in the structure of various point defects, dislocations and interstitial defects can serve as additional obstacles to the accumulation and agglomeration of radiation-induced defects in the structure, leading to softening of the surface layer of ceramics. Unlike nitride ceramics or spinel-type ceramics, ZrO_2_ ceramics are highly resistant to amorphization as a result of irradiation, which leads to fewer changes in strength properties. In this case, the formation of an impurity substitutional phase in the structure, which leads to a decrease in the degree of polymorphic transformations, as well as to a decrease in the degree of swelling of the crystal lattice, can also have a positive effect on the resistance to softening of the damaged layers of ceramics.

**Figure 5:**
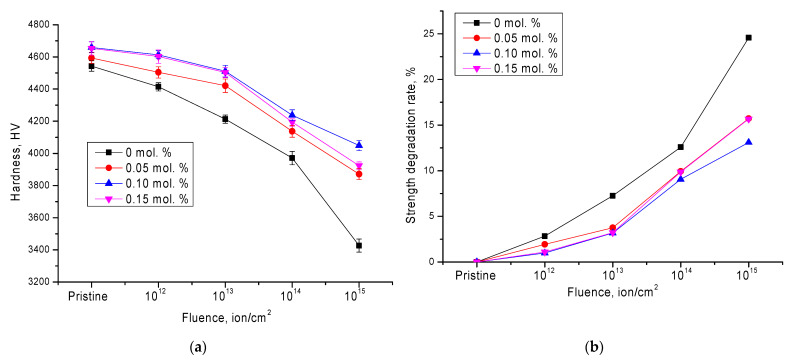
(**a**) Dependence of hardness change on irradiation fluence; (**b**) dependence of change in degree of hardness degradation on irradiation fluence.

Figure 5 shows the results of changing the strength characteristics—in particular, the hardness and degradation degree—depending on irradiation fluence. According to the data obtained, doping leads to an increase in hardness of the initial samples by 1.1–2.4%, depending on the MgO concentration. This increase is due to a change in the structural parameters and an increase in the density of ceramics.

For irradiated samples without doping, the change in hardness values can be divided into two stages, characterized by a different degree of degradation and a decrease in hardness. The first stage is typical for irradiation fluences of 10^12^–10^14^ ion/cm^2^, for which the decrease in hardness values can be described by a linear dependence. As shown in the data presented, in the case of undoped ceramics, for which the degree of polymorphic transformations is maximum at an irradiation fluence of 10^13^ ion/cm^2^, a sharp decrease in strength is observed. Furthermore, there is an increase in the damaged layer softening degree above 15%, with an increase in irradiation fluence observed. At the same time, an increase in the irradiation fluence up to 10^15^ ion/cm^2^ leads to a sharp deterioration in hardness indicators, and the degradation degree is more than 23%.

The addition of a dopant and the subsequent formation of the Zr_0.9_Mg_0.1_O_2_ phase, which leads to a decrease in the degree of polymorphic transformations, results in a lower softening degree and an increase in resistance to hardness degradation. At the same time, for irradiation fluences of 10^12^–10^13^ ion/cm^2^, the decrease in hardness is no more than 3–4%. An increase in irradiation fluence to 10^14^–10^15^ ion/cm^2^ leads to an increase in disordering degree to 12–15%, while for the original undoped ceramics, the maximum disordering degree for this fluence was more than 20%.

**Figure 6:**
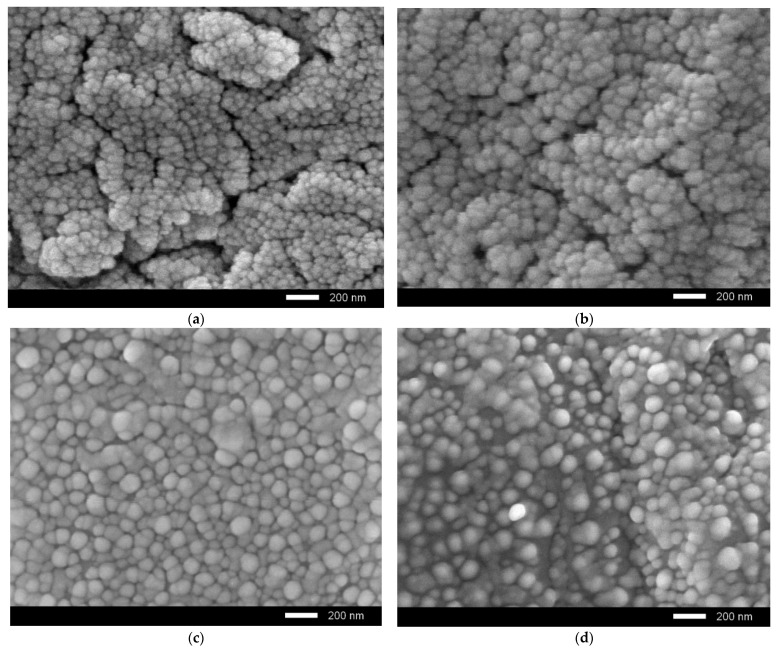
Results of morphological studies of ceramics irradiated with a fluence of 10^15^ ion/cm^2^: (**a**) pristine sample; (**b**) 0.05 mol.%; (**c**) 0.10 mol.%; (**d**) 0.15 mol.%.

Figure 6 shows the results of morphological studies of ceramics after irradiation with the maximum irradiation fluence, to determine the degradation degree of the near-surface layer.

As seen in the data presented, an increase in the dopant concentration leads to an enlargement of grains and an increase in the homogeneity degree of their sizes. At the same time, in the case of the initial samples irradiated with a fluence of 10^15^ ion/cm^2^, delamination and the formation of microcracks and pores are observed in the near-surface layer; this indicates a high concentration of overstresses and deformations, leading to cracking and a decrease in the strength and crack resistance of ceramics. In turn, doping leads to an increase in stability, as well as a decrease in microcracks. It should be noted that at a dopant concentration of 0.10 mol.%, the formation of microcracks and pores is not observed in the near-surface layer, which indicates a high resistance of ceramics to irradiation and degradation.

In general, it can be noted that, according to the data obtained, the doping of ZrO_2_ ceramics with MgO at a concentration of 0.05–0.10 mol.% leads to an increase in radiation resistance due to the formation of a substitution phase. The presence of this phase leads not only to an increase in radiation resistance, but also to the formation of defect regions in the structure. As is known, in the case of irradiation with heavy ions or neutron radiation, the main factor affecting the decrease in strength properties are point defects, the concentration of which increases with the radiation dose. At the same time, one of the factors for restraining the decrease in strength properties is the use of various dopants [34,35], which make it possible to reduce the rate of migration of point defects along the structure by changing the dislocation density or additional grain boundaries.

## 4. Conclusions

As a result of this study, it was found that the formation of Zr_0.9_Mg_0.1_O_2_ impurity phase in the structure of ZrO_2_ ceramics leads not only to a decrease in the degree of polymorphic transformations but also to an increase in resistance to radiation damage and a decrease in strength properties.

During determination of the most effective dopant concentration, it was found that the addition of MgO with a content of 0.10 mol.% is the most optimal concentration, leading to the formation of Zr_0.9_Mg_0.1_O_2_ phase, the content of which does not exceed 3–4%. At the same time, an increase in Zr_0.9_Mg_0.1_O_2_ phase in the structure of the initial ceramics leads to the appearance of additional distortions and tensile stresses in the crystal lattice, which have a negative effect on the change in the structure as a result of accumulation of radiation defects.

The results obtained indicate that the addition of MgO in concentrations of 0.05–0.10 mol.% significantly increases the resistance of ZrO_2_ ceramics to radiation damage and subsequent polymorphic transformations, thereby increasing the stability and radiation resistance of ceramics.

## Data Availability

The data presented in this study are available on request from the corresponding author.

## References

[1] Zhao H., Wu L. (2020). Forecasting the non-renewable energy consumption by an adjacent accumulation grey model. J. Clean. Prod..

[2] Ding S., Tao Z., Zhang H., Li Y. (2022). Forecasting nuclear energy consumption in China and America: An optimized structure-adaptative grey model. Energy.

[3] Vo D.H., Vo A.T., Ho C.M., Nguyen H.M. (2020). The role of renewable energy, alternative and nuclear energy in mitigating carbon emissions in the CPTPP countries. Renew. Energy.

[4] Ingersoll D.T., Carelli M.D. (2020). Handbook of Small Modular Nuclear Reactors.

[5] Lobach K.V., Kuprin O.S., Sayenko S.Y., Voyevodin V.M., Kolodiy I.V. (2020). Research and Development of Novel Materials for Accident Tolerant Fuel Cladding of Nuclear Reactors. East Eur. J. Phys..

[6] Kadyrzhanov K.K., Tinishbaeva K., Uglov V.V. (2020). Investigation of the effect of exposure to heavy Xe22+ ions on the mechanical properties of carbide ceramics. Eurasian Phys. Tech. J..

[7] Khafizov M., Pakarinen J., He L., Hurley D.H. (2019). Impact of irradiation induced dislocation loops on thermal conductivity in ceramics. J. Am. Ceram. Soc..

[8] Trukhanov A.V., Kozlovskiy A.L., Ryskulov A.E., Uglov V.V., Kislitsin S.B., Zdorovets M.V., Tishkevich D.I. (2019). Control of structural parameters and thermal conductivity of BeO ceramics using heavy ion irradiation and post-radiation annealing. Ceram. Int..

[9] Averback R.S., Ehrhart P., Popov A.I., Sambeek A.V. (1995). Defects in ion implanted and electron irradiated MgO and Al_2_O_3_. Radiat. Eff. Defects Solids.

[10] Kotomin E.A., Kuzovkov V.N., Popov A.I., Vila R. (2016). Kinetics of F center annealing and colloid formation in Al_2_O_3_. Nucl. Instrum. Methods Phys. Res. Sect. B Beam Interact. Mater. At..

[11] Khafizov M., Pakarinen J., He L., Hurley D.H. (2021). Study of the radiation disordering mechanisms of AlN ceramic structure as a result of helium swelling. J. Mater. Sci. Mater. Electron..

[12] Ahmad S.A., Husnain G., Ajmal M., Song P. (2021). Irradiation with phosphorus ions modifies the structure and tunable band-gap of a hexagonal AlN thin film. Appl. Phys. A.

[13] Gnesin G.G. (1996). Development of nonoxide ceramics based on silicon carbide and nitride. Powder Metall. Met. Ceram..

[14] Talmy I.G., Zaykoski J.A., Opeka M.M. (2010). Synthesis, processing and properties of TaC–TaB2–C ceramics. J. Eur. Ceram. Soc..

[15] Zhang X., Hilmas G.E., Fahrenholtz W.G. (2009). Densification and mechanical properties of TaC-based ceramics. Mater. Sci. Eng. A.

[16] Wei K., He R., Cheng X., Zhang R., Pei Y., Fang D. (2014). Fabrication and mechanical properties of lightweight ZrO2 ceramic corrugated core sandwich panels. Mater. Des..

[17] Altunal V., Guckan V., Ozdemir A., Kurt K., Ekicibil A., Yegingil Z. (2020). Investigation of luminescence properties of BeO ceramics doped with metals for medical dosimetry. Opt. Mater..

[18] Ionescu E., Linck C., Fasel C., Müller M., Kleebe H.J., Riedel R. (2010). Polymer-derived SiOC/ZrO2 ceramic nanocomposites with excellent high-temperature stability. J. Am. Ceram. Soc..

[19] Du Z., Zeng X.M., Liu Q., Lai A., Amini S., Miserez A., Gan C.L. (2015). Size effects and shape memory properties in ZrO2 ceramic micro-and nano-pillars. Scr. Mater..

[20] Liu Y., Zhu Y., Shen T., Chai J., Niu L., Li S., Wang Z. (2021). Irradiation response of Al2O3-ZrO2 ceramic composite under He ion irradiation. J. Eur. Ceram. Soc..

[21] Ivanov I.A., Alin M., Koloberdin M.V., Sapar A., Kurakhmedov A.E., Kozlovskiy A.L., Uglov V.V. (2021). Effect of irradiation with heavy Xe22+ ions with energies of 165–230 MeV on change in optical characteristics of ZrO2 ceramic. Opt. Mater..

[22] Alin M., Kozlovskiy A.L., Zdorovets M.V., Uglov V.V. (2021). Comprehensive study of changes in the optical, structural and strength properties of ZrO2 ceramics as a result of phase transformations caused by irradiation with heavy ions. J. Mater. Sci. Mater. Electron..

[23] Alin M., Kozlovskiy A.L. (2020). Study of structural changes in ZrO2 ceramics irradiated with heavy ions of Kr15+ with an energy of 147 MeV. Phys. Sci. Technol..

[24] Zu Y., Chen G., Fu X., Luo K., Wang C., Song S., Zhou W. (2014). Effects of liquid phases on densification of TiO2-doped Al2O3–ZrO2 composite ceramics. Ceram. Int..

[25] Deng W., Li Y. (2019). High-temperature electrical properties of polycrystalline MgO-doped ZrO2. Mater. Res. Bull..

[26] Sarkar D., Mohapatra D., Ray S., Bhattacharyya S., Adak S., Mitra N. (2007). Synthesis and characterization of sol–gel derived ZrO2 doped Al_2_O_3_ nanopowder. Ceram. Int..

[27] Singh B.K., Roy H., Mondal B., Roy S.S., Mandal N. (2018). Development and machinability evaluation of MgO doped Y-ZTA ceramic inserts for high-speed machining of steel. Mach. Sci. Technol..

[28] Medvedev P.G., Frank S.M., O’Holleran T.P., Meyer M.K. (2005). Dual phase MgO–ZrO2 ceramics for use in LWR inert matrix fuel. J. Nucl. Mater..

[29] Dittmer M., Yamamoto C.F., Bocker C., Rüssel C. (2011). Crystallization and mechanical properties of MgO/Al2O3/SiO2/ZrO2 glass-ceramics with and without the addition of yttria. Solid State Sci..

[30] Wang S., Zhai Y., Li X., Li Y., Wang K. (2006). Coprecipitation Synthesis of MgO-Doped ZrO2 Nano Powder. J. Am. Ceram. Soc..

[31] Belío-Reyes I.A., Bucio L., Cruz-Chavez E. (2009). Phase composition of ProRoot mineral trioxide aggregate by X-ray powder diffraction. J. Endod..

[32] Auerkari P. (1996). Mechanical and Physical Properties of Engineering Alumina Ceramics.

[33] Jagielski J., Thomé L., Nowicki L., Turos A., Gentils A., Garrido F., Aubert P. (2005). Ion irradiation of ceramic oxides: Disorder production and mechanical properties. Nucl. Instrum. Methods Phys. Res. Sect. B Beam Interact. Mater. At..

[34] Costantini J.M., Beuneu F., Gourier D., Trautmann C., Calas G., Toulemonde M. (2004). Colour centre production in yttria-stabilized zirconia by swift charged particle irradiations. J. Phys. Condens. Matter.

[35] Savoini B., Ballesteros C., Santiuste J.M., González R., Popov A.I., Chen Y. (2001). Copper and iron precipitates in thermochemically reduced yttria-stabilized zirconia crystals. Philos. Mag. Lett..

